# Editorial: Enzyme-based theranostics

**DOI:** 10.3389/fbioe.2023.1280647

**Published:** 2023-08-29

**Authors:** Ru Huang, Bowen Shu

**Affiliations:** ^1^ State Key Laboratory of Digital Medical Engineering, School of Biomedical Engineering, Hainan University, Haikou, China; ^2^ Key Laboratory of Biomedical Engineering of Hainan Province, One Health Institute, Hainan University, Haikou, China; ^3^ Dermatology Hospital, Southern Medical University, Guangzhou, China

**Keywords:** enzyme, theranosctics, enzyme targets, CRISPR, nanozyme

Enzymes are a class of substance having specifically catalytic function. In organisms, there are a variety of enzymes, which participate in almost all life activities, such as growth, development, metabolism, reproduction, aging, etc. Furthermore, a large number of enzymes maintain excellent catalytic function *in vitro*. The unique catalytic efficiency and specificity render them to be useful tools in diagnosis, gene engineering, and biomedicine. Nowadays, the advance of molecular biotechnology and structure biology leads to the discovery of new enzymes, as well as the rational evolution of the existing enzymes, and thus promoting enzyme-based theranostic technology. This Research Topic aims at providing and sharing new idea and technology involved in enzyme or enzyme mimics for precise diagnosis and therapy.

In a healthy situation, the abundance and activity of enzymes in living organisms are maintained at a relatively stable level. Relatively, abnormal enzyme abundance and activity are closely related to many diseases. Therefore, many enzymes have been confirmed to be ideal target for disease diagnosis and therapy. Liang et al. reviewed the physiological mechanism and function of deubiquitinases (DUBs), a class catalase that can remove ubiquitin from substrate protein and thereby upregulate protein level, and involve in the proliferation, apoptosis, metastasis and autophagy of tumor cells. The review also highlights the application of the small molecule inhibitors of DUBs in tumor treatment. Luque-Campos et al. reviewed the roles of several key enzyme regulators in mitochondrial dysfunction of different neurodegenerative disorders. Understanding the regulation mechanism and research status of these enzymes could help further develop combination diagnostic and therapy methods to tackle diseases such as cancer, Alzheimer’s disease, etc.

Enzymes derived from the defense system of procaryotic organisms, typically such as restriction endonuclease (REase), played vital roles in gene engineering (Pingoud et al., 2014). In recent years, clustered regularly interspaced short palindromic repeats and its associated enzyme (CRISPR/Cas) system revolutionized gene editing and regulation technology (Wang and Doudna, 2023) due to its transcendental specificity, simplicity and flexible programmability, and being recognized by the *2020 Nobel Prize in Chemistry* for the “*the invention of CRISPR technology for genome editing.*” Beyond that, CRISPR/Cas has also spawned the next-generation diagnostic technology (Weng et al., 2023). Jiang et al. reported a novel method named Bio-SCAN V2 that utilizes dead Cas9 (dCas9) without DNA cleavage activity as a ligand-responsive signal probe and realizes rapid and visual detection of theophylline on lateral flow test strip. Wang et al. leveraged another CRISPR associated enzyme—Argonaute (Ago) for specific signal amplification in sensitive microRNA detection. As the CRISPR toolbox continues to expand, these effective and flexible tools could further improve gene diagnostics and therapeutics.

Generalized enzymes include many kinds of entities with catalytic activity. Besides protein, some nucleic acids (DNA or RNA) having ability to catalyze the cleavage of nucleic acids or induce redox reaction are named as DNAzyme/ribozyme (Chang et al., 2021). Similarly, some inorganic nano-materials have been found to have peroxidase or catalase-like activity and named as nanozyme (Wu et al., 2021). Li et al. constructed a Fe-based metal-organic framework material exhibiting catalase-like activity to consume excess H_2_O_2_ in tumor microenvironment, thereby improving the efficacy of photodynamic and photothermal therapy. Compared to natural enzymes, nanozymes are easier to prepare and mass-produce, and transport. Moreover, the activity of nanozymes can be flexibly adjusted by changing the type and proportion of the components, the structure, as well as the surface modification (Tang et al., 2021).

Cell signaling pathways are complicate and the function of enzyme could be affected by multiple factors, thus a single biomarker is difficult to pinpoint a particular disease. That is, why many cancer biomarkers are tested to monitor the progression and treatment of cancer rather than early screen and detection (Crosby et al., 2022). Alternatively, combinational diagnosis/therapy could be a more ideal pathway for precise theranostics. In the future, more efforts should be devoted to the discovery and clarification of the related mechanism of biomarkers. As a class of diagnosis and therapy tools, enzymes also can have great application potential. The discovery and application of CRISPR/Cas is a great example showcasing how important enzymes are to the development of biological diagnostics and therapeutics. Up to now, nanozymes have also developed into a large family. The development of enzyme screening and manual construction technology for enzymes will further broaden its application prospect in the field of theranostics. We express our gratitude to all contributing authors and welcome more contributions from scholars in this area either fundamental science or applications in the future.
